# Analyzing Nicotinamide Adenine Dinucleotide Phosphate Oxidase Activation in Aging and Vascular Amyloid Pathology

**DOI:** 10.3389/fimmu.2017.00844

**Published:** 2017-07-31

**Authors:** Helena Radbruch, Ronja Mothes, Daniel Bremer, Stefanie Seifert, Ralf Köhler, Julian Pohlan, Lennard Ostendorf, Robert Günther, Ruth Leben, Werner Stenzel, Raluca Aura Niesner, Anja E. Hauser

**Affiliations:** ^1^Department of Neuropathology, Charité – Universitätsmedizin Berlin, Berlin, Germany; ^2^German Rheumatism Research Center (DRFZ), A Leibniz Institute, Berlin, Germany; ^3^Immune Dynamics, Deutsches Rheuma-Forschungszentrum (DRFZ), A Leibniz Institute, Berlin, Germany; ^4^Immune Dynamics, Charité – Universitätsmedizin Berlin, Berlin, Germany

**Keywords:** aging, NADPH oxidases, microglia, astrocytes, Alzheimer’s disease, cerebral amyloid angiopathy

## Abstract

In aging individuals, both protective as well as regulatory immune functions are declining, resulting in an increased susceptibility to infections as well as to autoimmunity. Nicotinamide adenine dinucleotide phosphate (NADPH) oxidase 2-deficiency in immune cell subsets has been shown to be associated with aging. Using intravital marker-free NAD(P)H-fluorescence lifetime imaging, we have previously identified microglia/myeloid cells and astrocytes as main cellular sources of NADPH oxidase (NOX) activity in the CNS during neuroinflammation, due to an overactivation of NOX. The overactivated NOX enzymes catalyze the massive production of the highly reactive ${\text{O}}_{2}^{-},$ which initiates in a chain reaction the overproduction of diverse reactive oxygen species (ROS). Age-dependent oxidative distress levels in the brain and their cellular sources are not known. Furthermore, it is unclear whether in age-dependent diseases oxidative distress is initiated by overproduction of ROS or by a decrease in antioxidant capacity, subsequently leading to neurodegeneration in the CNS. Here, we compare the activation level of NOX enzymes in the cerebral cortex of young and aged mice as well as in a model of vascular amyloid pathology. Despite the fact that a striking change in the morphology of microglia can be detected between young and aged individuals, we find comparable low-level NOX activation both in young and old mice. In contrast, aged mice with the human APP^E693Q^ mutation, a model for cerebral amyloid angiopathy (CAA), displayed increased focal NOX overactivation in the brain cortex, especially in tissue areas around the vessels. Despite activated morphology in microglia, NOX overactivation was detected only in a small fraction of these cells, in contrast to other pathologies with overt inflammation as experimental autoimmune encephalomyelitis (EAE) or glioblastoma. Similar to these pathologies, the astrocytes majorly contribute to the NOX overactivation in the brain cortex during CAA. Together, these findings emphasize the role of other cellular sources of activated NOX than phagocytes not only during EAE but also in models of amyloid pathology. Moreover, they may strengthen the hypothesis that microglia/monocytes show a diminished potential for clearance of amyloid beta protein.

## Introduction

Following the free-radical theory of aging, reactive oxygen species (ROS) are massively produced, e.g., >100 nM H_2_O_2_, and attack their targets in the organism randomly, indiscriminative and cumulative, thus, generating oxidative distress (1). Oxidative distress is a general term for the dysfunction of signaling and defense mechanisms based on ROS.

As several ROS are highly reactive, their localization is crucial for the resulting effects: on the one hand, ROS act as specific signaling molecules intracellularly at ~1–10 nM concentration, on the other hand, they constitute effective extracellular host defense mechanisms at concentrations over 100 nM. Since the organism has efficient mechanisms to neutralize high ROS concentrations, e.g., via glutathione peroxidase or superoxide dismutase (2), these phenomena are physiological and can be summarized under the term “oxidative eustress” (1). The enzymes of the nicotinamide adenine dinucleotide phosphate (NADPH) oxidase family, consisting of NOX1, NOX2 (phox), NOX3, NOX4, DUOX1, DUOX2 (3), are central players leading both to oxidative eustress and distress. Their activation catalyzes the oxidative burst when abundant highly reactive ${\text{O}}_{2}^{-}$ is produced by oxidation of molecular oxygen. Under enzymatic catalysis, ${\text{O}}_{2}^{-}$ reacts with various small molecules leading to massive ROS production. When this massive ROS production exceeds the capacities of antioxidant defense mechanisms of the tissue or when extracellular highly reactive ROS species such as H_2_O_2_ enter the cells *via* peroxiporins such as AQP8 (4), oxidative distress occurs, leading to tissue dysfunction and damage. It is assumed that oxidative distress responsiveness is linked to aging. However, this hypothesis is mainly funded on genetic studies (5), which allow conclusions on the expression levels, but not on the actual activation of these central enzymes.

Here, we focus on NADPH oxidase enzyme activation levels in the brain, as this organ is especially vulnerable to oxidative distress. There is evidence that the aged brain is more susceptible to injuries (6). This is mainly attributed to a so-called activated basal state of low-grade chronic inflammation that has been called “inflamm-aging” (7). Low-grade inflammation in aging is also associated with microgliosis; however, the function of microglia in this process is highly discussed as studies using cell morphology, protein expression, cellular dynamics, or *ex vivo* cytokine production could detect age-dependent differences (8). No information on the age-dependent oxidative distress levels and their cellular sources *in situ* are available.

In this study, we determine NADPH oxidase activation levels in the context of amyloid pathology, in order to investigate age-dependent, immune-mediated tissue damaging mechanisms in neurodegenerative diseases. We hypothesize that an age-dependent dysregulation of immune responses in the CNS contributes to neuroinflammatory processes associated with neurodegeneration. ROS have been implicated in mediating age-dependent changes and promoting age-dependent neurodegeneration (9). Recently, oxidative distress has been regarded as an early sign of Alzheimer’s disease (AD) pathophysiology, although the source of ROS and the mechanisms how amyloid peptides (Aβ) influence oxidative distress have not been adequately investigated (10). Subunits of NOX2 are upregulated in patients with mild cognitive impairment compared to normal age-matched controls. During disease progression, a further increase of the cytosolic subunits p67phox, p47phox, and p40phox could be detected in the CNS tissue. In addition, there was a robust correlation between NOX subunit expression and the individual’s cognitive status (11). Together, this suggests that increases in NOX activity participate in early AD pathogenesis and contribute to AD progression due to massive ROS production initiated by NADPH oxidases, activating signaling pathways leading to neuronal excitotoxicity and glial cell-mediated inflammation (12).

Although many studies claim to analyze NOX activity, only limited information can be gained based on the analysis of subunit expression levels. The quantification of ROS levels in tissue has been widely used. However, the highly diffusive nature of ROS does not allow to draw conclusions on their origin in tissues. Up to now, neither the catalytic activity of NOX enzymes leading to oxidative distress nor their cellular sources could be tracked *in vivo*. We therefore use our previously published marker-free method of intravital fluorescence lifetime imaging (FLIM) of NAD(P)H (13) to analyze the distribution and cellular source of NADPH oxidase activation (14, 15). Since activated NOX enzymes are membrane-bound and can be detected by FLIM, this technique allows for an unambiguous identification of the cellular source of massive ROS production.

## Materials and Methods

### Two-Photon Laser-Scanning Microscopy

For intravital imaging, a frontoparietal cranial window preparation was performed and the dural layer was removed according to previous publications (16). Both fluorescence intensity and FLIM experiments were performed as previously described with a two-photon laser-scanning microscope based on a commercial scan head (TriMScope, LaVision BioTec, Bielefeld, Germany). All images are acquired in 30–150 µm depth within the frontoparietal cortex (*z*-step = 2 µm). Ten imaging fields per mouse were acquired, and the fields of vision with the highest NOX enzyme activation in every group were included into analysis. The detection of the fluorescence signals was accomplished either with photomultiplier tubes in the ranges 460 ± 30, 525 ± 25, 593 ± 20 nm or with a 16-channel parallelized TCSPC detector (FLIM-X_16_, LaVision BioTec, Bielefeld, Germany) in the range 460 ± 30 nm. The excitation of NADH and NADPH [hereafter collectively referred to as NAD(P)H] was performed at 760 nm (detection at 460 ± 30 nm). Dextran–rhodamine (detection at 593 ± 20 nm) and EGFP (detection at 525 ± 25 nm) were excited at 850 or 880 nm. For intensity and FLIM, we used an average maximum laser power of 8 mW. The experimental parameters for FLIM were 80 ps histogram bin [for NAD(P)H-FLIM] and maximum acquisition time for a 512 × 512 image was 5 s to record a fluorescence decay stack. The time-window in which the fluorescence decays were acquired was set to 9 ns.

### Data Analysis

Fluorescence lifetime imaging data analysis was carried out with self-written software, as previously described (14, 15). We used a bi-exponential decay function (Eq. 1):

(1) $$I_{\text{NAD}(\text{P})\text{H}}(t)=\varepsilon +a_{1}\cdot e^{-t/\tau_{1}}+a_{2}\cdot e^{-t/\tau_{2}}$$

with ε the background, the 1-indexed term of the sum representing the fluorescence decay of free NADH and NADPH, and the 2-indexed term representing the fluorescence decay of enzyme-bound NAD(P)H. The fluorescence lifetime τ_1_ [free NAD(P)H] is 400–450 ps, while the fluorescence lifetime τ_2_ of NAD(P)H bound to metabolic enzymes has an average of ~2,000 ps and of 3,650 ps if bound to an enzyme of the NOX family. We focused in this study on the fluorescence lifetime τ_2_, of the enzyme-bound NAD(P)H. As previously described (14), we calculated the area of NOX activation within a τ_2_ image as the percentage of pixels having fluorescence lifetimes between 3,300 and 3,900 ps as compared to the total number of pixels displaying fluorescence lifetimes between 0 and 10,000 ps.

In order to quantify microglial ramification in young and old Iba1:GFP mice from immunofluorescence data, we used our previously published algorithm based on discrete Fourier coefficients (17). Briefly, single microglia cells were segmented from the green channel of the immunofluorescence data acquired in the cortex of healthy 6 or 20 months old Iba1:GFP mice. Their shape was approximated by overlapping circles—the first centered in the center of the cell and the others centered on the periphery of the previous circle. Each layer of circles is mathematically characterized by a scalar parameter called Fourier coefficient. The first Fourier coefficient represents the position of the cell, the second defines its dimensions by approximating it with a sphere, and the next coefficients define the degree of ramification of the cellular processes.

Statistical analysis and graphical presentation was performed with GraphPad Prism 4 (GraphPad Software, USA) and OriginPro (OriginLab, USA). Results are shown as mean values from the analyzed imaging fields (±SD) per group.

### Mice

APP^E693Q^ i.v. labeled with sulforhodamine 101 (for astrocyte staining *in vivo*) or APP^E693Q^:Iba-1-EGFP mice (18, 19) hemizygous for both genes were generated and maintained on a C57BL/6J background. Littermates with wild-type murine APP were used as controls. Groups of *n* ≥ 3 mice were used in all *in vivo* experiments and also for postmortem histological analysis, *n* ≥ 3 mice per group were used. Young mice were 6 months old and aged mice were between 18 and 24 months old at time of analysis (in APP mutant and wild-type group; individual mouse age listed in Table 1). Animals were group housed in standard cages under pathogen-free conditions on a 12-h light/dark cycle with food and water *ad libitum*. All animal experiments were performed in accordance to the national animal protection guidelines approved by the regional veterinary office for health and social services in Berlin (LaGeSo Berlin).

**Table 1:** Overview of NOX activation from individual mice analyzed.

Mouse ID	Age (months)	NOX activation (%)
WT	6	1.7
Young 1		
WT	6	1
Young 2		
WT	6	0.6
Young 3		
WT	24	0.3
Old 1		
WT	20	0.7
Old 2		
WT	18	1.5
Old 3		
WT	20	1.8
Old 4		
APP	6	0.8
Young		
APP	24	4.3
Old 1		
APP	24	4.2
Old 2		
APP	20	4.2
Old 3		
APP	18	5.2
Old 4		
APP	18	5.4
Old 5		

*1–4 imaging fields were analyzed per mouse. NOX activation represents mean values*.

### Murine Tissue Processing and Immunofluorescence Histology

After each intravital microscopy experiment, the brains of the mice were prepared for immunofluorescence by perfusion with 4% paraformaldehyde. The tissue was embedded in Tissue Tek (Sakura), frozen in a methylbutane/dry ice mixture into 10- or 30-µm sections using a cryostat. Sections were stained with goat anti-EGFP (Rockland, conjugated at the DRFZ to Alexa^®^488), mouse anti-GFAP-Alexa^®^488 (eBioscience, Germany), rabbit anti-Noxo-1 (Novus Biologicals, Germany), or goat anti-p47 (Abcam, Germany). Secondary antibodies used were donkey anti-rabbit Alexa^®^647 (Life Technologies, Germany) or donkey anti-goat-Alexa^®^647. Slides were analyzed on a Zeiss LSM 710 confocal system. Co-localization was analyzed by standardized background subtraction and subsequently multiplying the Noxo1 and GFAP channel using with Fiji Software.

### Human Tissue Processing and Immunofluorescence Histology

Brain samples were obtained from the Department of Neuropathology, Charité – Universitätsmedizin Berlin. We included only anonymized individuals with cerebral amyloid angiopathy (CAA). The study was approved by the local ethics committee under the number EA1/078/16. Paraffin-embedded brain tissues were sectioned at 4 µm with a microtome (Microm HM330, GMI), followed by deparaffinization and rehydration in a decreasing ethanol series. For immunofluorescence staining, sections were blocked with 10% normal serum (NS) for 1 h. After washing, sections were incubated with primary antibodies mouse a-hu HLA-DR 1:100 (Dako), goat a-hu p47phox 1:100 (Abcam), rabbit a-GFAP (DAKO) 1:2,000, and mouse a-oxidized phospholipids at 1:50 (clone E06, Avanti Polar Lipids) with 2% NS. Secondary antibodies (goat a-ms-Alexa^®^546 and chicken a-goat-Alexa^®^594, donkey a-rabbitAlexa^®^488) were diluted 1:500 with 2% NS and added to the sections. Nuclei were stained with DAPI (Sigma) at a 1:1,000 dilution in phosphate-buffered saline. Slides were analyzed on a Zeiss LSM 710 confocal system.

## Results

### Differences in the Morphology, but Similar NADPH Oxidase Activation Levels in Cortical Microglia of Young and Old Mice

**Figure 1:**
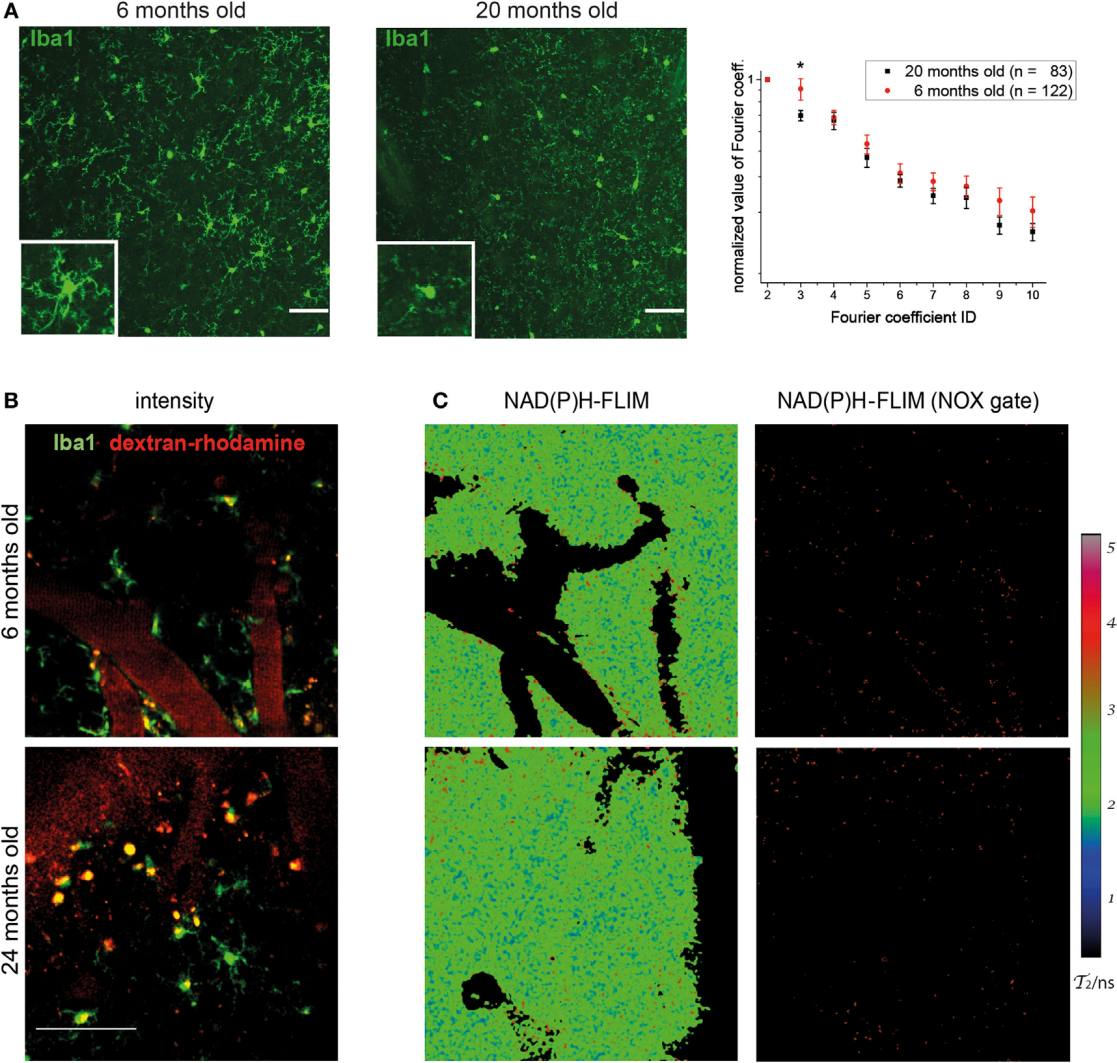
Morphology of cortical microglia and nicotinamide adenine dinucleotide phosphate (NADPH) oxidase activation in young and old healthy mice. **(A)** Iba-1:EGFP reporter mice were stained with anti-EGFP and 30-µm thick sections were analyzed of frontoparietal cortex by confocal microscopy. Representative immunofluorescence images (maximum intensity projections) from the neocortex of a 6-month-old (left) and 20-month-old (right) mouse indicate the differences in morphology of Iba1-EGFP^+^ cells (green). *Scale bar*: 50 µm. In the graph, the normalized values of the 3^rd^ to the 10^th^ Fourier coefficients, calculated as described in the manuscript, indicate that the ramification of the microglial processes is higher in young (*n* = 4) than in old healthy mice (*n* = 4). **(B)** Representative images of PMT-based detection of fluorescence signals and **(C)** enzyme-bound NAD(P)H-fluorescence lifetime imaging (FLIM) maps (τ_2_-maps) in a young (6 months; upper row) and old (24 months; lower row) mouse. *Scale bar*: 50 µm. The τ_2_-maps of the left column show the false color-encoded fluorescence lifetime τ of enzyme-bound NAD(P)H at each recorded pixel of the image. NAD(P)H bound to metabolic enzymes are depicted in blue and green (τ_2_ between 1 and 3 ns), whereas NADPH bound to activated NOX enzymes appears in red (τ_2_ between 3.3 and 3.9 ns, “NOX only” gate) is displayed in the right column of **(B)**.

When analyzing the morphology of microglia in Iba-1-EGFP reporter mice by immunofluorescence microscopy in various age cohorts, we noticed striking differences between young and old individuals. While the majority of microglia in mice at the age of 6 months displayed a ramified, surveilling morphology in the cortex, we noted that those cells in mice between 18 and 24 months of age had short, blunt processes, suggesting a more activated phenotype (Figure 1A). Using our previously published algorithm based on discrete Fourier coefficients (17), we found a higher ramification of the microglial processes in young mice (*n* = 122 cells) than in old mice (*n* = 83 cells) as indicated by all Fourier coefficients up to the 10^th^ rank, especially by the 3^rd^ Fourier coefficient (graph in Figure 1A).

The detection of these morphological differences in microglia between various young and aged mice prompted us to investigate whether they are actually accompanied by differences in the functionality of these cells. This was performed by making use of our previously established method of *in vivo* FLIM (20). The method uses a time-correlated single-photon counting device in order to detect the endogenous fluorescence lifetime of NAD(P)H, as a differential indicator for the activity of metabolic enzymes as well as for NOX enzyme activation in tissue. In combination with PMT-based detection of fluorescence signals (Figure 1B), we are able to allocate NOX enzyme activation to certain neocortical regions and cell types. By applying these techniques in the neocortex of Iba1-EGFP mice injected with dextran–rhodamine, we neither detected significant differences in the overall activation state of NOX-dependent enzymes nor an increase in NOX enzyme activation specifically in Iba-1^+^ cells in aged mice (Figure 1C). Taken together, this indicates a comparable level of metabolic activity of NOX enzymes in young and old mice.

### Locally Elevated NOX Activity in the Cortex of Aged APP^E693Q^ Mice and Oxidized Phospholipids in Patients with CAA

Next, we aimed to investigate microglia activation in an aging-associated disease. We chose an age-dependent model of congophilic Aβ CAA. In this model, transgenic mice (APP^E693Q^ mice) overexpress E693Q-mutated human APP under the control of the neuron-specific Thy1 promoter element (19, 21, 22). Mice with the APPE693Q mutation exhibit glial reaction and neuronal apoptosis in certain areas of the brain without extensive presence of Aβ plaques in the parenchyma. However, APP^E693Q^ mice display fibrillary Aβ at the vessel walls leading to an amyloid angiopathy (starting at an age of 9 months), causing hemorrhagic strokes and dementia in aged mice. In order to monitor microglia activity *in vivo*, we crossed the APP^E693Q^ mice with Iba1-EGFP mice (18).

**Figure 2:**
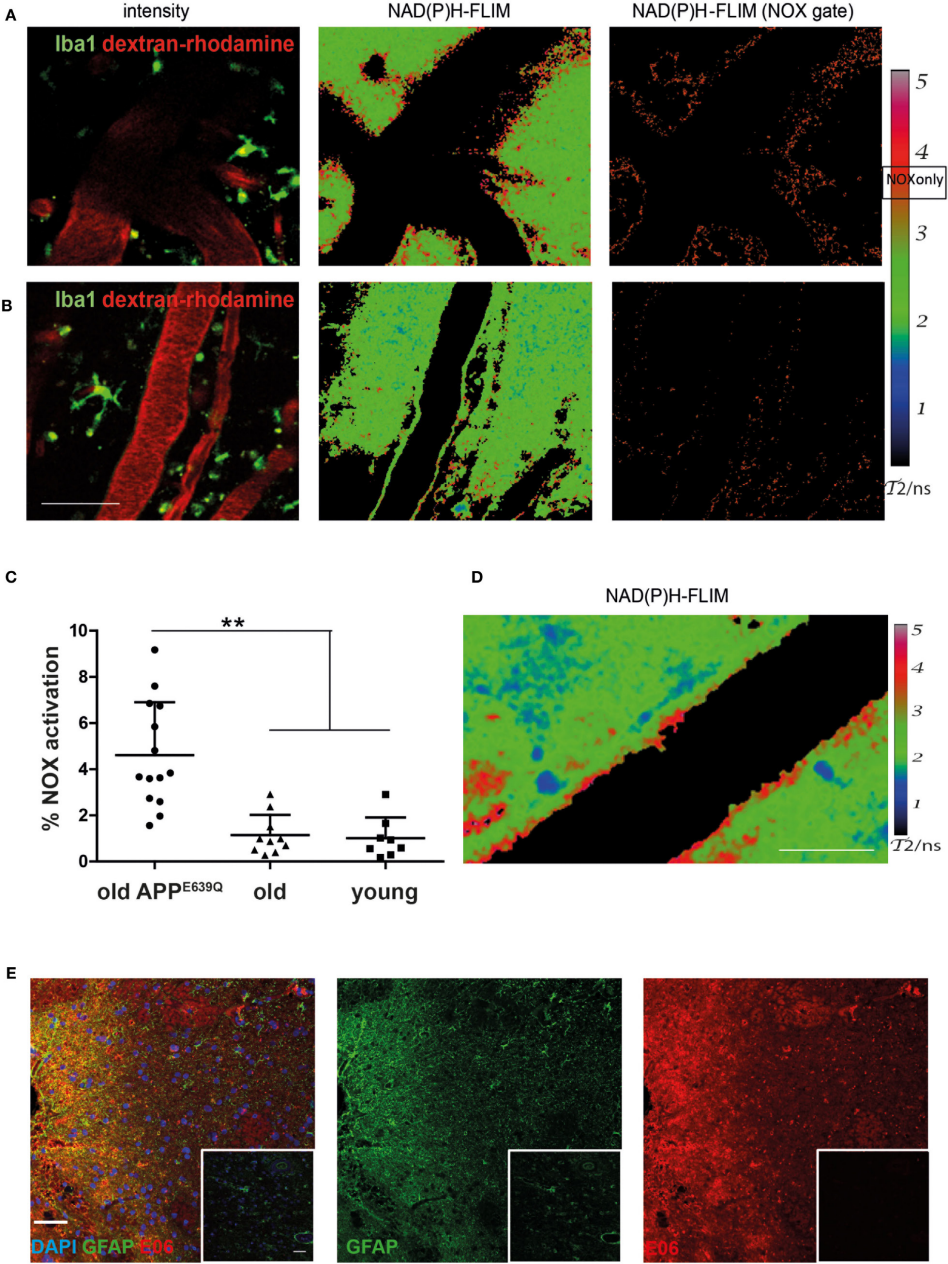
Elevated NOX activity in the cortex of aged APP^E693Q^ mice. Representative images of areas with elevated **(A)** and low **(B)** NOX enzymes activation are both found in old APP^E693Q^ mice with established cerebral amyloid angiopathy (CAA). PMT-based detection of fluorescence signals [left column **(A,B)** dextran–rhodamine in red and iba1^+^ cells in green] in a 24-month-old APP^E693Q^ mouse. *Scale bar*: 50 µm. The corresponding τ_2_-maps of the middle column show the false color-encoded fluorescence lifetime τ of enzyme-bound NAD(P)H at each recorded pixel of the image. NAD(P)H bound to metabolic enzymes are depicted in blue and green (τ_2_ between 1 and 3 ns), whereas nicotinamide adenine dinucleotide phosphate (NADPH) bound to activated NOX appears in red (τ_2_ between 3.3 and 3.9 ns, “NOX only” gate) is displayed in the right column of **(A,B)**. **(C)** Quantification of the NOX activation area within, i.e., ratio of the area of NOX only gate to the total tissue area, 4.9 ± 2.0% (*n* = 14 fields of view of 5 mice with 2–4 fields of view per mouse) in APP^E693Q^ mice compared to 1.1 ± 0.7% (*n* = 10 fields of view of 4 mice with 1–4 fields of view per mouse) and 1.0 ± 0.9% (*n* = 8 fields of view of 3 mice with 2–3 fields of view per mouse) in healthy controls (old and young, respectively). At least 3 mice per group and 2–4 imaging fields per mouse were analyzed. For statistic evaluation, we applied the ANOVA test (**p < 0.01). **(D)** The τ2 NAD(P)H-fluorescence lifetime imaging (FLIM) map shows a specific bilayered pattern in the proximity of the vasculature of APP^E693Q^ mice. *Scale bar*: 25 µm. **(E)** Representative immunofluorescence image within the cortex of patients with CAA showing strong reactivity with the E06 antibody recognizing oxidized phospholipids in areas with astrogliosis (stained with anti-GFAP antibody) *Scale bar*: 50 µm, compared to normal appearing tissue from patients with CAA (shown in insets). *Scale bar*: 50 µm.

We analyzed the activation of NOX enzymes in the frontoparietal neocortex by intravital microscopy. For visualization of the vasculature, mice were injected with dextran–rhodamine prior to imaging. NAD(P)H-FLIM revealed heterogeneous local elevations in NOX activity in APP^E693Q^ mice, and an overall elevation of NOX enzyme activity in the tissue was apparent. This effect could be mainly attributed to focal spots with elevated NOX activity (Figure 2A), which occurred near areas of normal appearing tissue with no elevation in NOX activity. Age-matched control mice did not exhibit tissue regions of elevated NOX activity (Figure 2B), as reflected in the quantification of the overall percentage of NOX-signal present in the tissue (Figure 2C). The mean area of NOX enzyme activation was 4.9 ± 2.0% (*n* = 5 mice) in APP^E693Q^ mice older than 20 months. In contrast, aged-matched wild-type controls had only a mean of 1.1 ± 0.9% (*n* = 4 mice) NOX enzyme activation area, a value comparable with young wild-type mice: mean = 1.0 ± 0.9% (*n* = 3 mice) and with young APP^E639Q^ mice: mean = 0.8 ± 0.6% (*n* = 1 mouse) (Table 1). Regarding the distribution and range, we found that elevated NOX activation was enhanced in the immediate vicinity of blood vessels of APP^E693Q^ mice. The activation appeared in a layered pattern around the vessels: besides the endothelium being strongly activated, we also detected other activated structures in the tissue neighboring the endothelia; frequently, these structures were found to run parallel to the vessels (Figure 2D). Having demonstrated the overactivation of NOX, we next addressed the question to what extent this NOX activity affects the CNS tissue in terms of oxidative distress. We therefore stained CNS tissue of patients with CAA by histology, using an antibody (E06) that recognizes the phosphocholine headgroup of oxidized phospholipids (23) in combination with an anti-GFAP antibody labeling astrocytes. Consistent with our results regarding elevated NOX activation in the APP^E693Q^ mouse model, we found a strong focal presence of oxidized phospholipids, localized in areas with an astrogliosis as displayed by GFAP immunoreactivity, compared to normal appearing tissue in patients with CAA (Figure 2E, *n* = 4).

### Minor Role of Microglia As Cellular Sources of Activated NOX in Aged APP^E693Q^ Mice and in Patients with CAA

**Figure 3:**
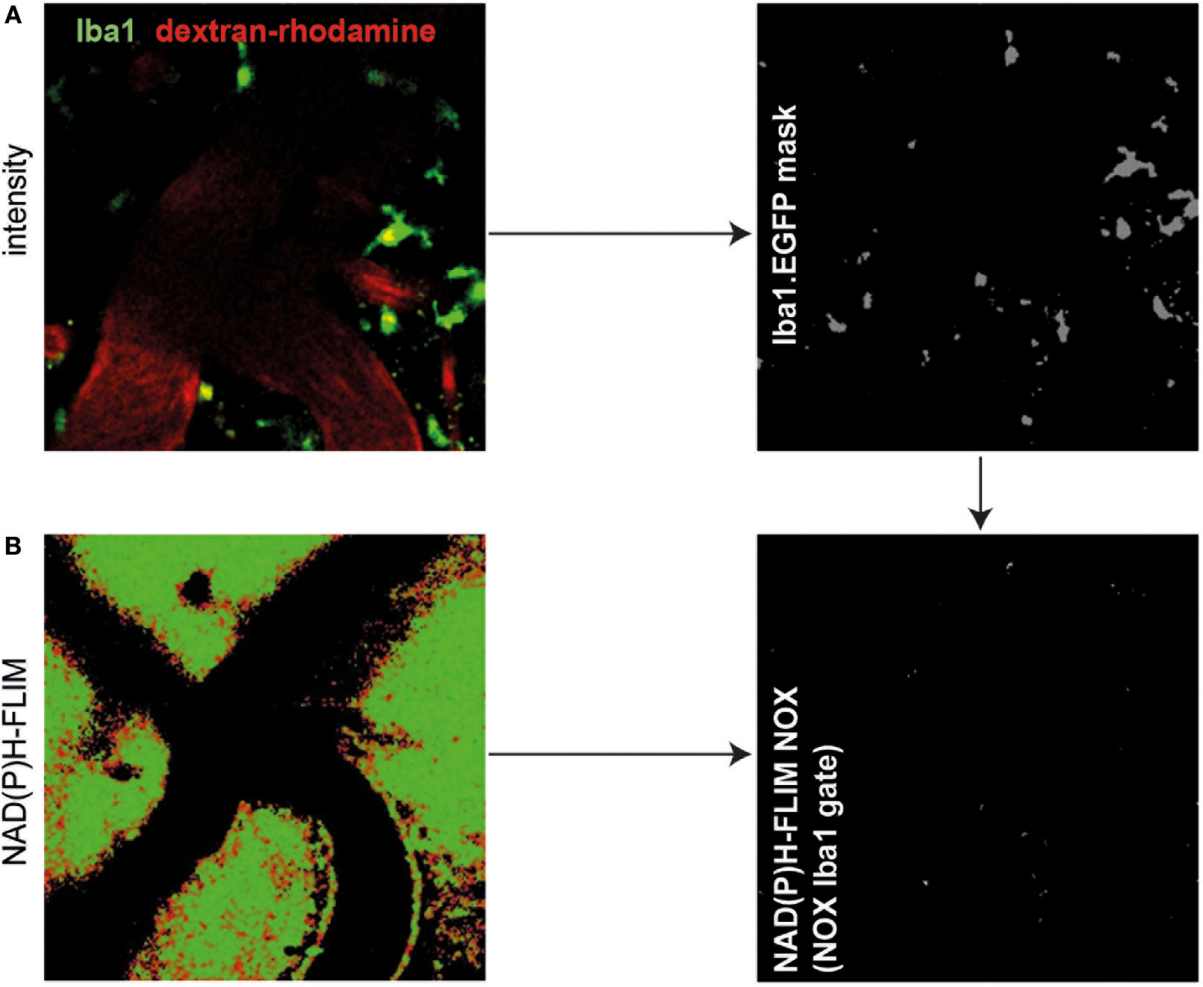

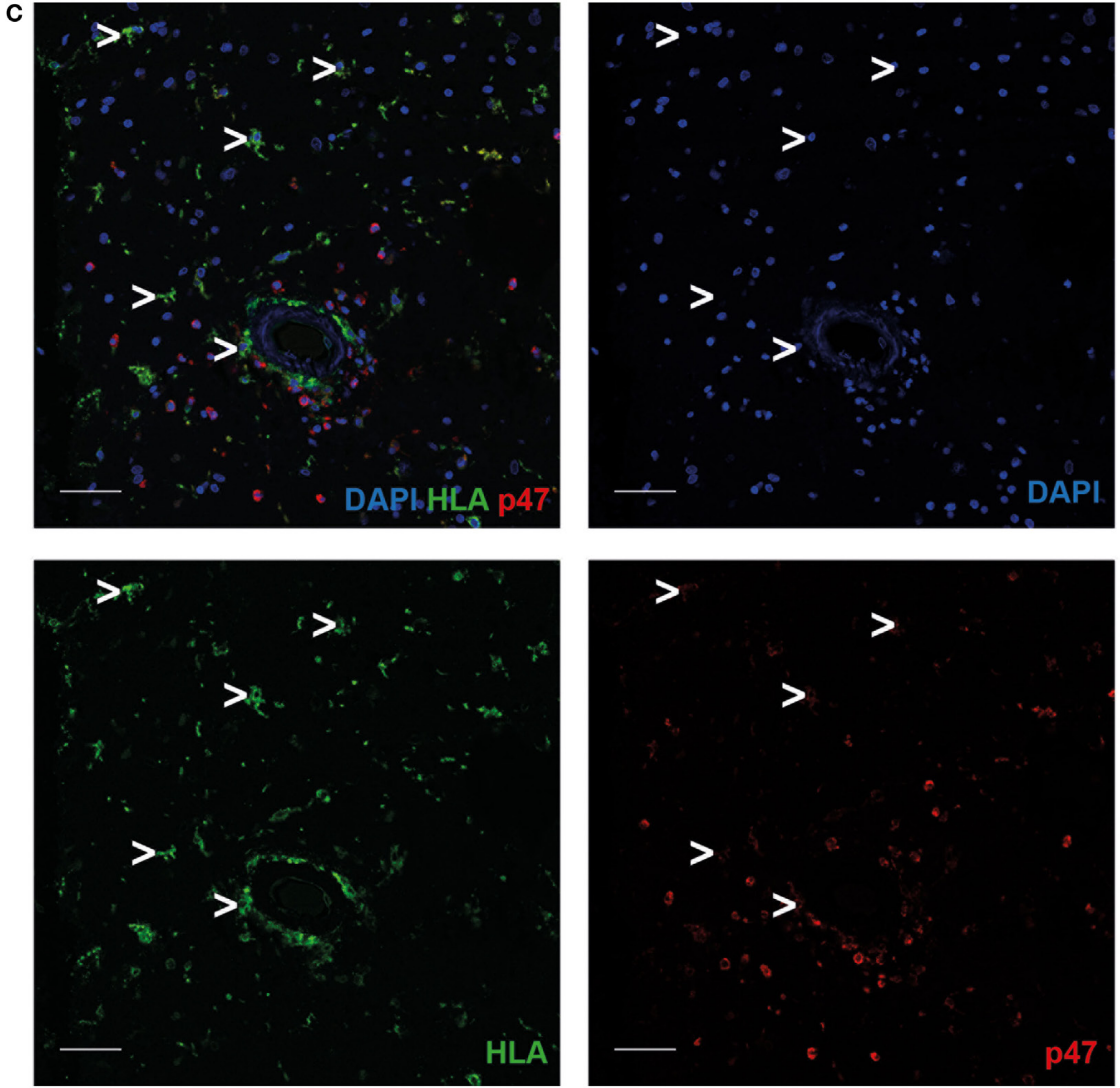
Minor role of microglia as cellular sources of activated NOX in APP^E693Q^ mice and patients with cerebral amyloid angiopathy (CAA). Fluorescence intensity images of the cortex of a Iba1-EGFP-APP^E693Q^ mouse **(A)** and corresponding τ_2_ NAD(P)H-fluorescence lifetime imaging (FLIM) maps of the whole tissue area **(B)** were compared to determine the contribution of microglia to the NOX activation signal. We performed an overlay of the two images, as in **(A,B)**, and analyzed the NAD(P)H-FLIM signal at the areas of the Iba-1^+^ cells (green). The τ_2_ NAD(P)H-FLIM images depict the normalized area of NOX activation in relation to the total cellular area in the Iba-1^+^ cell subsets: mean 1.6 ± 0.8% out of 3 mice; (2–4 fields of view per mouse). *Scale bar*: 50 µm. **(C)** Representative immunofluorescence image within the cortex of patients with CAA showing enriched p47 at the membrane of blood-derived innate immune cells but not in cells with microglial morphology (white arrowheads) (*n* = 4 patients. *Scale bar*: 50 µm).

In order to further identify the cellular source of NOX activity in heterozygous APP^E693Q^ mice, we analyzed aged APP^E693Q^:Iba1-EGFP mice by intravital NAD(P)H-FLIM. Microglia were identified based on their EGFP fluorescence and vessels identified by the presence of dextran–rhodamine (Figure 3A, left panel). By using the EGFP-signal for masking the image of the NAD(P)H-fluorescence lifetime data (Figure 3A, right panel), we were able to determine the levels of NOX activation specifically in microglia (Figure 3B). This “gating” strategy revealed that only a minor fraction (mean = 1.6 ± 0.8%; *n* = 3 mice) of the total NOX enzymes activity actually originated from microglia, whereas the major fraction arouse from other cellular sources in the parenchyma. Moreover, when we analyzed the activation state of the microglia population, we found NOX enzyme activity in only ~2% of these cells (mean = 1.7 ± 1.4%; *n* = 3 mice, see Table 1). Taken together, these results indicate a minor relevance of microglia in the generation of ROS in the APP^E693Q^ mouse model and imply other, yet undefined, cells as main contributors.

Similar to our results regarding microglial NOX activation in the APP^E693Q^ mouse model, we found only low expression levels of p47 (a cytosolic subunit present in all NOX enzymes) in cells with microglial morphology as compared to blood-derived immune cells such as neutrophil granulocytes or macrophages/monocytes (Figure 3C, *n* = 4) in patients with CAA.

### Major Contribution of Astrocytes to Activated NOX in Aged APP^E693Q^ Mice

**Figure 4:**
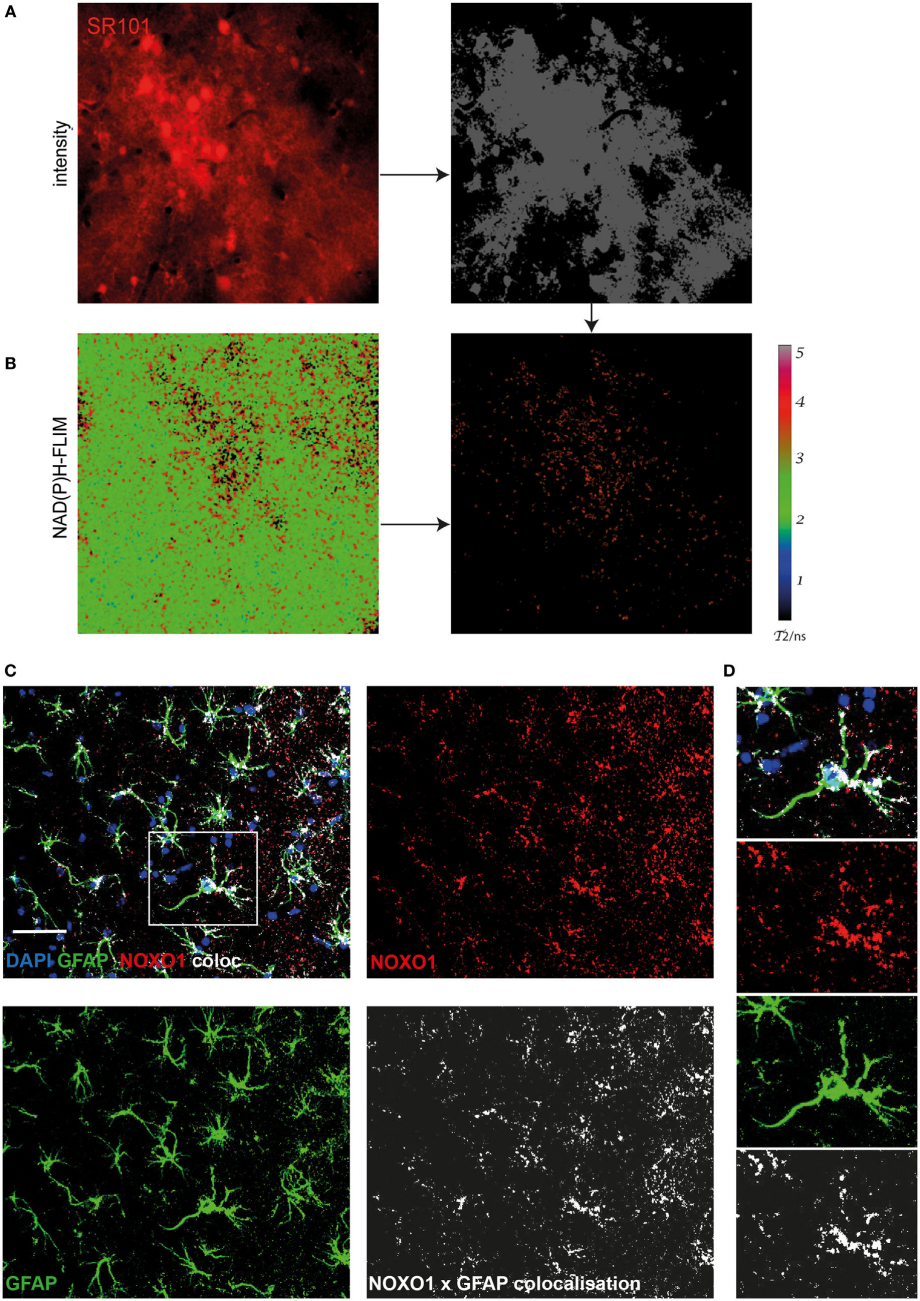
Astrocytes are major cellular sources of activated NOX in APP^E693Q^ mice. Similar to Figure 3, fluorescence intensity images of the cortex of a APP^E693Q^ mice labeled with sulforhodamine 101 (SR101) **(A)** and corresponding τ_2_ NAD(P)H-fluorescence lifetime imaging (FLIM) maps of the whole tissue area **(B)** were correlatively analyzed to measure the contribution of SR101^+^ cells (mainly astrocytes) to the NOX activation signal. We performed an overlay of the two images and analyzed the NAD(P)H-FLIM signal at the areas of SR101 signal (red). The τ_2_ NAD(P)H-FLIM images depict the normalized area of NOX activation in relation to the total cellular area in the SR101 labeled cell subsets: mean 37.9 ± 2.1% out of 2 mice; with 2–3 fields of view per mouse. *Scale bar*: 50 µm. **(C)** Representative immunofluorescence image within the cortex of a 20-month-old APPE693Q mouse indicating the distribution of Noxo1 (subunit of NOX1, red) and GFAP signal (green). Nuclei are stained with DAPI (blue). *Scale bar*: 50 µm. White box marks inset displayed in **(D)**.

Using immunofluorescence histology and intravital NAD(P)H-FLIM, we previously found NOX1-expressing astrocytes as contributors to NOX enzyme overactivation in the context of chronic neuroinflammation (17). Based on that, we hypothesized that astrocytes may also contribute to NOX enzyme activation observed in APP^E693Q^ mice. In order to identify further cellular sources of NOX in the cortex of these mice, we performed intravital NAD(P)H-FLIM after local staining with sulforhodamine 101 (SR101). Astrocytes were identified based on their SR101 fluorescence (Figure 4A, left panel), which was used to mask the corresponding NAD(P)H-fluorescence lifetime maps (Figure 4B, left panel) and, thus, to determine the NOX activation levels in these cells (Figure 4B, right panel). By “gating” the NAD(P)H-FLIM maps in this way, we identified SR101 labeled cells (astrocytes) to be major cellular contributors to the NOX activation signal in the cortex of aged APP^E693Q^ mice (mean = 37.9 ± 2.1%; *n* = 2 mice).

In order to verify this finding using immunofluorescence histology, we stained brain cortex sections from aged APP^E693Q^ mice with antibodies against the Noxo1, a membrane-bound subunit of NOX1. While NOX2 is mainly expressed in phagocytic cells, other NOX enzymes prevail in other cell types (3, 17). Consistent with our intravital data, we could detect an enriched membrane-bound Noxo1 signal, co-localizing with processes of astrocytes (Figures 4C,D). Taken together, our data suggest that the observed bi-laminar NOX activation pattern in APP^E693Q^ mice consists of an inner endothelial NOX activation and an outer astrocytic NOX activation layer.

## Discussion

Age-related changes in immune responses, commonly known as immunosenescence, are increasingly moving in the focus of immunological research. Immunosenescence is a multifactorial phenomenon caused, among other factors, by the diminished potential of hematopoietic stem cells to self-renew (24), age-dependent impairment of antigen-presenting cells (25), a reduction of T cell (26) and B cell repertoire and numbers (27), causing a decline in adaptive immunity (28). A reduction in numbers of phagocytes has been described in aged individuals (29), along with an impaired functionality, which has been suggested to reflect the adaptation to age-associated changes in their environment (30).

On a molecular level, an age-related deficiency in NOX2 has been recently shown to result in a reduced suppressive function in aged CD8^+^ regulatory T cells (31), leading to inflammation and tissue destruction. Whether changes in NOX activity also occur in other immune subsets during aging, especially in phagocytic cells, which are known to express NOX2 at high levels, is not known. By analyzing the changes in fluorescence lifetime of NAD(P)H as a means to analyze NOX activation *in vivo*, we recently demonstrated that NOX enzyme overactivation in cerebral phagocytes contributes to oxidative distress associated with chronic neuroinflammation. Here, we compared the activation of microglia in young versus aged mouse cohorts. We found a striking difference in microglia morphology between the two groups. While the majority of those cells in young mice showed a ramified morphology typical of a surveilling behavior, microglia in old mice typically exhibited an activated phenotype characterized by shorter processes. Our findings are in line with a previous report in mice (32), describing similar changes in morphology. Moreover, a recent study has confirmed similar age-dependent changes in human microglia (33).

However, none of the previous studies functionally analyzed microglia in different age cohorts *in vivo*. We chose to take NOX enzyme activation as a functional readout for microglia activation. Despite the obvious differences in morphology, we could not detect differences between young and old individuals, when comparing NOX activation by NAD(P)H-FLIM in the cerebral cortex of mice. In old and young healthy mice, there was also no elevated NOX enzyme activation in the CNS tissue, arguing against a chronic low-grade tissue inflammation, which has been associated with immunosenescence, at least in microglia.

Next, we aimed to investigate NOX activation in cerebral phagocytes during age-related neurodegenerative disease. AD, a fatal neurodegenerative condition, is one of the most common causes of dementia in aged. Major pathological changes are the deposition of extracellular amyloid β (Aβ) peptide plaques and formation of neurofibrillary tangles, which consist of the microtubule protein tau, in the CNS tissue. In the patients, neuronal dysfunction progressively leads to cognitive impairment and loss of memory. While extensive research has been performed to elucidate the role of amyloid deposits in AD, the role of inflammation in the brain has only recently become appreciated. Microglia are in the focus of interest, especially during disease progression. Aβ not only aggregates in neuronal parenchyma but can also accumulate on blood vessel walls, this condition is named CAA and can cause hemorrhagic strokes and dementia. We found elevated levels of oxidized phospholipids by immunofluorescence in cerebral tissue of patients with CAA, suggesting ongoing tissue damage by oxidative distress in areas with pronounced astrogliosis. We used a mouse model of CAA in order to analyze NOX enzyme activity *in vivo*. In line with the cerebral angiopathy prevailing in this mouse model, we found a strong activation of NOX in tissue areas around the blood vessels in a typical bilayered pattern. However, our analyses did not show significant elevated NOX enzyme activity in microglia, but rather in other CNS cells such as astrocytes. We could confirm this hypothesis by performing intravital NAD(P)H-FLIM in APP^E639Q^ mice in which astrocytes were labeled by sulforhodamine 101, in which we found that more than a third of overactivated NOX enzymes are associated with astrocytes. By histology, the expression of the NOX1-subunit Noxo1 was partly localized in GFAP^+^ cells, underlining the role of astrocytes as contributors to the elevated NOX enzyme activity in the tissue. Further cellular sources of NOX enzyme activation may include endothelial cells that have been shown to express NOX4 (34) or pericytes and vascular smooth muscle cells. However, these results extend our previous findings, which identified astrocytes as main source of oxidative stress in chronic neuroinflammation, especially when peripheral immune cells were not abundant in the CNS parenchyma during later stages of the disease (17). It should be noted that the level of NOX activation measured in the CAA model is around three times lower than in experimental autoimmune encephalomyelitis (EAE) (14) and even more in a murine glioblastoma model (16), where up to one-third of the CNS tissue shows NOX activity. This finding supports the idea of a rather low-grade chronic process occurring during neurodegenerative diseases, in contrast to an overt inflammatory response. However, a continued—rather low-level—ROS production over time can exceed the anti-oxidative capacity of the tissue and lead to a slow, but progressive tissue destruction. Hence, we expect the ROS production in CAA to be in the pathologic range, i.e., >100 nM, but rather low as compared to the ROS concentrations found in chronic neuroinflammation of ~200 μM (14). Intravital NAD(P)H-FLIM reveals parallels in the mechanisms between autoimmune-mediated chronic neuroinflammation and primary neurodegenerative diseases. Our data point to astrocytes as important cellular players, which are able to maintain a state of local low-grade inflammation in the CNS tissue *via* extracellular ROS production. It should be emphasized at this point that by detecting NOX activity, our method is not suitable for measuring intracellular ROS production (~1–10 nM), e.g., resulting from free radicals generated in the mitochondrial respiratory chain. Rather, it focuses on the massive (physiologic and pathologic) production of extracellular ROS, for which the NADPH oxidase family plays a central role.

Interestingly, the specific pattern of NOX activation we found in the tissue of the CAA model differed from the one we had previously detected in lesions of mice with EAE or glioma. A specific NOX enzyme signal was present in the endothelial layer as well as in the vicinity of the vasculature. However, the latter did not originate from microglia in aged APP^E693Q^ mice. There is increasing evidence in the literature that Aβ causes an impairment of microglia function (35). It remains to be tested whether our findings can be extended to other Aβ aggregation forms as seen, e.g., in plaques and to other microglial functions. In addition, it will be important to address the question if locally enhanced oxidative distress precedes the formation of Aβ aggregates in the tissue, or if it is a consequence of this process.

## Ethics Statement

This study was carried out in accordance with the recommendations of the Landesamt for Gesundheit and Soziales, Berlin, Germany. The protocol was approved by the Landesamt für Gesundheit und Soziales, Berlin, Germany.

## Author Contributions

DB, HR, JP, RN, RM, RL, and SS designed and performed research and analyzed data. RM, HR, RN, and AH wrote the manuscript. HR and AH initiated, organized, and supervised the project. SS provided animals and expertise in animal models with APP pathology. HR, RG, and AH provided expertise in mouse handling and intravital imaging. WS, RM, LO, JP, and AH collected the human samples and analyzed human data. RN, RK, DB, and RL developed algorithms and analyzed data.

## Conflict of Interest Statement

The authors declare that the research was conducted in the absence of any commercial or financial relationships that could be construed as a potential conflict of interest.
